# Intraoperative active and passive breaks during minimally invasive surgery influence upper extremity physical strain and physical stress response—A controlled, randomized cross-over, laboratory trial

**DOI:** 10.1007/s00464-023-10042-9

**Published:** 2023-04-21

**Authors:** Tessy Luger, Rosina Bonsch, Robert Seibt, Bernhard Krämer, Monika A. Rieger, Benjamin Steinhilber

**Affiliations:** 1 Institute of Occupational and Social Medicine and Health Services Research, Eberhard Karls University and University Hospital Tübingen, Wilhelmstraße 27, 72074 Tübingen, Germany; 2 Plastic, Reconstructive and Burn Surgery, Clinic for Hand, BG Clinic Tübingen, Schnarrenbergstraße 95, 72076 Tübingen, Germany; 3 Department of Gynecology and Obstetrics, University Hospital Tübingen, Calwerstraße 7, 72076 Tübingen, Germany

**Keywords:** Laparoscopy, Gynecology, Interruptions, Electromyography, Working posture

## Abstract

**Objective:**

Investigate the effect of passive, active or no intra-operative work breaks on static, median and peak muscular activity, muscular fatigue, upper body postures, heart rate, and heart rate variability.

**Background:**

Although laparoscopic surgery is preferred over open surgery for the benefit of the patient, it puts the surgeons at higher risk for developing musculoskeletal disorders especially due to the less dynamic and awkward working posture. The organizational intervention intraoperative work break is a workplace strategy that has previously demonstrated positive effects in small-scale intervention studies.

**Methods:**

Twenty-one surgeons were exposed to three 90-min conditions: no breaks, 2.5-min passive (standing rest) or active (targeted stretching and mobilization exercises) breaks after 30-min work blocks. Muscular activity and fatigue of back, shoulder and forearm muscles were assessed by surface electromyography; upper body posture, i.e., spinal curvature, by inclination sensors; and heart rate and variability (HRV) by electrocardiography. Generalized estimating equations were used for statistical analyses. This study (NCT03715816) was conducted from March 2019 to October 2020.

**Results:**

The HRV-metric SDNN tended to be higher, but not statistically significantly, in the intervention conditions compared to the control condition. No statistically significant effects of both interventions were detected for muscular activity, joint angles or heart rate.

**Conclusion:**

Intraoperative work breaks, whether passive or active, may counteract shoulder muscular fatigue and increase heart rate variability. This tendency may play a role in a reduced risk for developing work-related musculoskeletal disorders and acute physical stress responses.

**Supplementary Information:**

The online version contains supplementary material available at 10.1007/s00464-023-10042-9.

Work-related musculoskeletal disorders (WRMSD) are well recognized among the general working population. Also, among all surgeons, the prevalence of WRMSD is reported to be 19% [1]. In different studies, about 55% [2], 74% [3], 87% [4], or up to 88% [5] of the laparoscopic surgeons reported experiencing musculoskeletal symptoms or discomfort due to their work. Because of patient benefits laparoscopy is generally preferred over open surgery (e.g., lower infection rates, shorter recovery times) [6–8], particularly as a result of this also during the COVID-19-Pandemic [9]. However, laparoscopic procedures also carry risks for the surgeon in developing WRMSD [10], particularly due to table height, monitor position, and poor handling of instruments [11]. These risks embrace less dynamic working postures [12, 13], awkward body postures [13, 14], and higher activation levels of several upper extremity muscles [15].

Occupational ergonomics may counteract these risk factors [16]; however, applying such ergonomics in the operating theater is challenging, because of the safety of the patient that should not be endangered. Nevertheless, several studies have implemented and evaluated different interventions, including workplace interventions [17, 18] and work-organizational interventions [19–22]. Both types of interventions have been evaluated in both lab and field studies and provide promising results, namely decreased discomfort in back and neck [20, 21] and shoulders [19, 21, 22], decreased lower leg muscle loading [23], decreased stress-related cortisol in saliva [22], improved wrist posture [17, 18], and showed no significant influence on surgery duration [19, 22].

The work-organizational intervention of intraoperative work breaks is of particular interest, because it does not only target the laparoscopic surgeon, but all other medical staff as part of the surgical team as well [24]. The benefit of including both a principal and assisting surgeon in laparoscopic surgeries to encounter long periods of static posture was also concluded by Al-Hakim, Xiao [25]: “Having experienced assistant surgeons to accompany surgeons in long procedures help surgeons to take sometimes to exercise, leaving the assistant surgeon to perform surgical operations”. Various types and durations of intraoperative work breaks in laparoscopic surgery have been evaluated. For type, both passive [22] and active breaks [19–21, 26–28] have been studied compared to no breaks, but never all together in one study. Passive breaks are breaks within which workers rest; active breaks are breaks within which workers perform a non-work-related physical or cognitive activity [29]. For duration, implementation of 20-s [21], 1.5-min [19, 20], and 5-min breaks [22] every 20 to 40 min work have been investigated.

The objective of the present study is to examine whether muscular fatigue, static, median and peak muscular activity, upper body postures, heart rate, and heart rate variability are changed when comparing passive and active with no work breaks and active with passive work breaks. These are secondary outcomes of the study; the primary outcome subjective discomfort together with the remaining secondary outcomes [30] will be reported in another manuscript.

## Materials and methods

### Study design

This study is registered at ClinicalTrials.gov (No. NCT03715816) [31], received ethical approval by the local ethical committee of the Medical Faculty of the University and University Hospital Tübingen (no. 618/2018BO2), and followed the ethical standards of the Declaration of Helsinki [32]. This laboratory study is a controlled, randomized cross-over trial that was conducted from March 2019 to October 2020. The study included a control condition (without breaks) and two intervention conditions (passive and active breaks) and was not blinded. The order of the three conditions was assigned to the participants by drawing lots.

### Study sample

We used the equation of Kadam and Bhalerao [33] to calculate the required sample size. Maintaining a 5% level of significance and 80% study power and using the converted results of the primary outcome perceived discomfort from Dorion and Darveau [21] (i.e., 1.6857 effect size and 1.9337 pooled SD), a sample size of 21 was determined, excluding potential drop-outs.

The subjects, i.e., laparoscopic surgeons, were recruited by direct advertisement and internal email announcements at the Department of Women’s Health at the University Hospital Tübingen. Eligibility criteria were: minimum age 18, proficiency in German, experience with (simulated) laparoscopy, and ability to perform the PEG-transfer task within 3 min [34]. We recruited 25 surgeons; after drop-out of four surgeons (one: too little time; two: acute injury on back and clavicula; one: employment contract ended), the final study sample consisted of 21 surgeons, whose demographics and musculoskeletal status, collected during the first laboratory visit (see Experimental Procedure for details), are summarized in Table 1.

**Table 1:** Demographics and musculoskeletal discomfort status of the final sample (N = 21) provided as absolute number, mean ± SD, or relative number (%)

	Total (*N* = 21)	Men (*N* = 12)	Women (*N* = 9)
Primary job description	Surgeon (13); Assistant (3); Student (5)	Surgeon (6); Assistant (3); Student (3)	Surgeon (7); Student (2)
Experience (years)	8.5 ± 5.6	7.6 ± 4.4	9.8 ± 6.9
Experience (laparoscopies)	< 100 (3); 100–200 (1); > 200 (17)	< 100 (2); 100–200 (1); > 200 (9)	< 100 (1); > 200 (8)
Current laparoscopies (#/week)	6.7 ± 5.6	6.8 ± 5.5	6.6 ± 6.0
Age (years)	36.6 ± 9.7	35.5 ± 7.7	38.0 ± 12.2
Weight (kg)	76.1 ± 13.8	82.5 ± 13.6	67.7 ± 9.0
Height (cm)	179.4 ± 8.3	182.8 ± 7.9	175.0 ± 6.9
BMI (kg/m^2^)	23.5 ± 3.1	24.7 ± 3.6	22.0 ± 1.5
Smoking status (yes/no)	Yes (4); No (17)	Yes (4); No (8)	No (9)
Sport (hours/week)	2.8 ± 2.8	2.4 ± 3.2	3.4 ± 2.4
Handedness	Right (21)	Right (12)	Right (9)
Musculoskeletal discomfort in the past 12 months | 7 days (percentages of the sample that reported yes)^A^			
Neck	33.3% | 14.3%	33.3% | 8.3%	33.3% | 22.2%
Shoulder	28.5% | 9.5%	33.3% | 8.3%	22.2% | 11.1%
Elbow	9.5% | 9.5%	16.7% | 16.7%	0.0% | 0.0%
Hand/wrist	14.3% | 4.8%	16.7% | 8.3%	11.1% | 0.0%
Upper back	23.8% | 14.3%	16.7% | 8.3%	33.3% | 22.2%
Lower back	47.6% | 14.3%	50.0% | 16.7%	44.4% | 11.1%
Hip/upper leg	9.5% | 4.8%	8.3% | 8.3%	11.1% | 0.0%
Knee	4.8% | 0.0%	0.0% | 0.0%	11.1% | 0.0%
Ankle/foot	0.0% | 0.0%	0.0% | 0.0%	0.0% | 0.0%

A, Multiple answers possible

### Intervention

The simulation of laparoscopic work lasted 90 min, which approximates the average duration of laparoscopic surgeries of 75 min [35]. In a highly controlled environment, 2.5-min breaks were provided after 30- and 60-min work blocks. This duration, frequency, and timing are based on the positive findings reported by previous studies that investigated intraoperative breaks [19, 20, 22]. Two experimental conditions were performed including passive or active breaks, and one control condition was performed not including breaks. In line with its definition [29], participants were instructed to just rest while standing during the passive breaks. In this study, the participant was allowed to lay down the laparoscopic instruments. The active work breaks contained a standardized audio recording (transcribed protocol, see Supplemental Digital Content 1) with instructions to perform a set of exercises focusing on posture correction, normalization of tissue tension, mobilization of soft tissue, and relaxation [26]. The exercise protocol was designed by the authors (TL, BS) in collaboration with a physical therapist, and aimed to target body regions that are particularly affected by discomfort or complaints, i.e., the neck, shoulders, and lower back [25, 36, 37]. We did not control for the number of repetitions and range of motion of each exercise.

### Experimental procedure

Prior to the experiment, the participant visited the lab for a familiarization to the experiment. During this first visit, the participant was explained about the study and its goals, filled out all necessary forms for participation and informed consent, practiced all tasks as simulated during the three conditions, and was asked about demographics (see Table 1) and musculoskeletal status according to the German version [38] of the standardized Nordic Musculoskeletal Questionnaire [39]. The experimental set-up was individually adjusted [40]: the table height provided a ~ 105° elbow angle while holding the instrument in a ~ 60° downward angle in the simulation device relative to the lower arm; the monitor height provided a ~ 10° downward view to avoid neck extension [41]. After adjustment, individual floor-table and floor-monitor heights were note, which equaled 71.5 cm (SD 4.9) and 105.7 cm (SD 6.9), respectively. The familiarization trial and the three experimental and control conditions were performed on separate days.

The 90-min simulation consisted of several tasks performed in a Pelvic Trainer (Szabo, ID Trust Medical, Belgium). Each task required optics (Karl Storz 26003AA HOPKINS II Optik 0°, Karl Storz SE & Co. KG, Tuttlingen, Germany) and the bimanual handling of one or two out of three tools: 34-cm long laparoscopic Maryland bipolar forceps (Model No. 20 195-225, ERBE Elektromedizin GmbH, Tübingen, Germany); 33-cm long forceps with 1:2 teeth (RS225-595, RUDOLF Medical GmbH & Co. KG, Fridingen, Germany); 36-cm long forceps without teeth (33,321 KW, KARL STORZ SE & Co. KG, Tuttlingen, Germany). The laboratory’s illuminance and room temperature were regulated to be 5–9 lx and 24–26 °C, respectively.

**Fig. 1:**
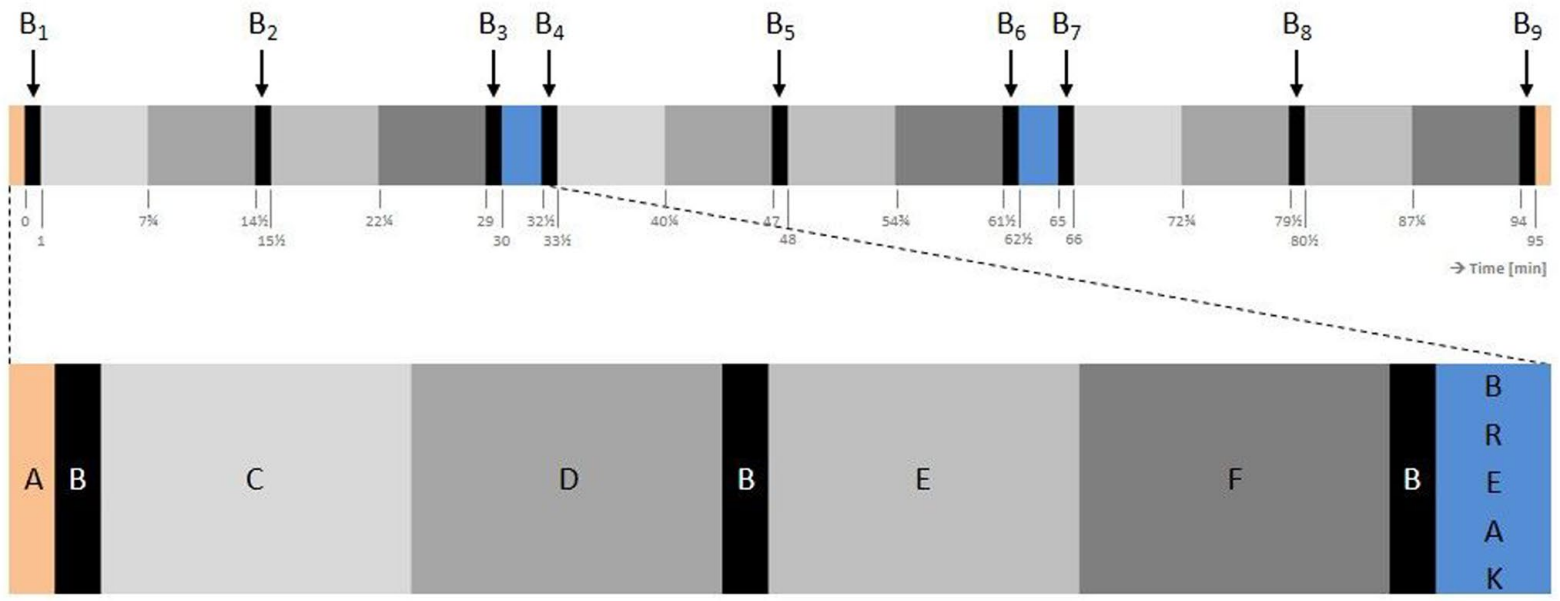
The time course of an experimental condition; in the control condition, the blue blocks (breaks) will vanish. Each block and letter is related to a task, including the hot-wire (**A**), peg-transfer (**B**), pick-and-place (**C**), pick-and-tighten (**D**), pick-and-thread (**E**) and pull-and-stick task (**F**). The arrows indicate the time points of recordings of muscle activity, upper body posture, and heart rate (B_1-9_) during the peg-transfer task (B)

**Fig. 2:**
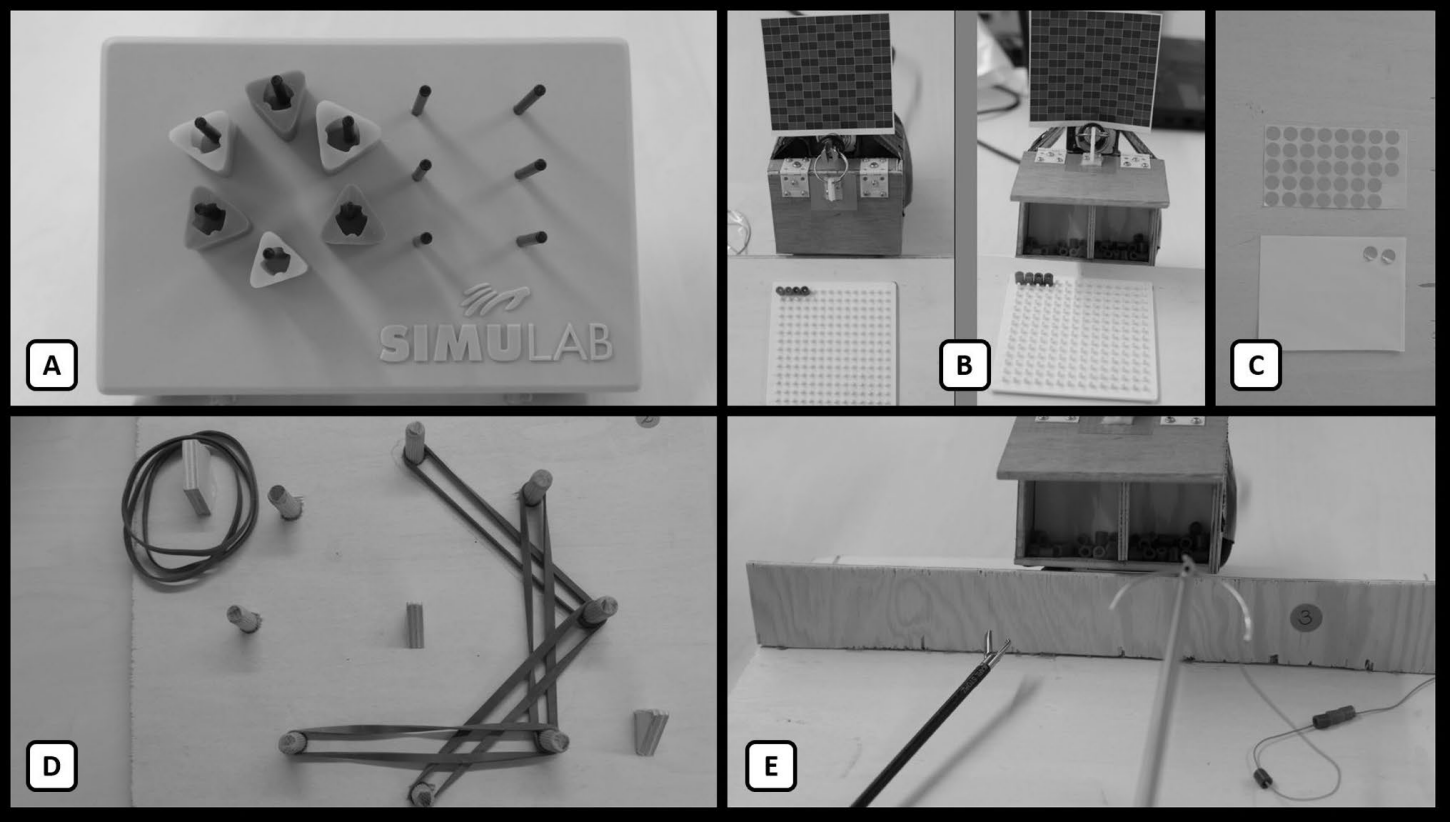
Visualization of the tasks that were performed as part of the experimental protocol: peg-transfer (**A**), pick-and-place (**B**), pull-and-stick task (**C**), pick-and-tighten (**D**), and pick-and-thread (**E**)

The 90-min simulation included three 30-min blocks, of which each block contained five tasks in a set order: peg-transfer, pick-and-place, pick-and-tighten, pick-and-thread, pull-and-stick. For a visualization of these task, see Fig. 1. Directly before and after the simulation, a hot-wire task was performed. A schematic overview of the tasks including breaks is provided in Fig. 2. During the pick-and-place and pick-and-thread tasks, a foot pedal was integrated with which a box was opened in order to pick the parts (i.e., beads). A comprehensive explanation of all six simulated tasks including the used instruments is provided in the study protocol [30].

### Data collection and data analysis

Data collection and analysis of the primary outcome perceived rating of discomfort and the secondary outcomes performance, workload, and subjective evaluation will be reported in another manuscript.

#### M﻿uscular activity and localized muscular fatigue

Electrical activity of seven muscles was recorded by surface electromyography (EMG) by placing two pre-gelled Ag/AgCl electrodes (42 × 24, Kendall™ H93SG ECG Electrodes, Covidien, Zaltbommel, Netherlands) in bipolar configuration (IED 25 mm) over the muscle belly [42, 43]. The ground electrode was placed over vertebra C7. The following muscles were recorded: erector spinae longissimus lumbalis (ES at vertebra L3, bilateral), trapezius descendens (TD, bilateral), deltoideus acromialis (DA, right), extensor digitorum (ED, right), flexor carpi radialis (FCR, right). EMG signals were collected using a data analyzer with data logger (PS11-UD, THUMEDI® GmbH & Co. KG, Thum, Germany; CMMR > 96 dB; overall effective sum of noise < 0.8 μV RMS; linearity ± 0.15 dB at 25–1100 Hz). EMG signals were differential amplified, analog filtered (high-pass filter, 4^th^ order, − 3 dB at 4 Hz; low-pass filter, 11th order, − 3 dB at 1300 Hz), and sampled (4096 Hz). Synchronous to data storage, EMG signals were real-time transformed into the frequency domain (1024-point Fast Fourier Transformation, Bartlett-window, 50% overlap), digitally high-pass filtered (11th order, − 3 dB at 16 Hz), and digitally average-filtered to remove powerline interferences (11th order, 50 Hz and first seven harmonics) by replacing it by the spectral values of a 4-Hz wide band around its center frequency by means of both spectral neighbors. Root-mean-square (RMS [μV]) and median power frequency (MPF [Hz]) were real-time calculated from the power spectrum and stored synchronously to the raw data by the PS11.

EMG was recorded continuously during 5-s maximal voluntary contractions (MVCs; see Supplemental Digital Content 2 for details about the MVCs) [44] and simulated laparoscopy. Two MVCs per muscle were performed with 1-min break in between prior to each condition. The maximal RMS of both MVCs per muscle was used to normalize the RMS of the experiment and expressed as percentage (%MVE). The 10th (static), 50th (median), and 90th percentiles (peak) of the RMS during B_1_, B_3_, B_4_, B_6_, B_7_, and B_9_ (cf. Figure 2) were calculated [45]. For localized muscular fatigue, we calculated the slope expressed as change per minute of the median RMS and MPF and plotted them against each other in joint analyses of the EMG spectrum and amplitude (JASA) [46]. In JASA plots, the lower right quadrant indicates muscular fatigue, reflected by an increased RMS and a decreased MPF.

#### Upper body postures

Six two-dimensional gravimetric inclination sensors (PS11; sample rate 8 Hz; resolution 0.1° and 125 ms in time; maximum static error 0.5°) were placed on the forehead, vertebrae T1, T10, L1, and L5, measuring flexion and lateral flexion angles with respect to the absolute perpendicular. The difference in flexion angles was used to calculate cervical lordosis or neck flexion (forehead–T1), thoracic kyphosis (T1–T10), and lumbar lordosis (L1–L5). The difference in lateral flexion angles was used to calculate neck lateral flexion (forehead–T1). The sensor on T10 was used to determine trunk flexion. The average angles during B_1_, B_3_, B_4_, B_6_, B_7_, and B_9_ (cf. Figure 2) were calculated.

#### Heart rate and variability

The electrical activity of the heart was recorded using electrocardiography (ECG) by two pre-gelled Ag/AgCl electrodes placed ~ 5 cm cranial and ~ 3 cm left-lateral from the distal end of the sternum and over the anterior to mid-axillary line at the fifth left rib. ECG signals were continuously recorded (sample rate 1000 Hz) and processed in real-time to calculate heart rate (HR [bpm]) and interbeat intervals (IBI [ms]). The IBI timeseries were checked for artifacts and erroneous intervals were excluded and replaced by polynomial interpolation (2^nd^ order) using MATLAB R2020a (The MathWorks Inc., Natwick, MA, USA). The corrected IBI timeseries were processed with Kubios HRV (Standard V3.3.1, Biosignal Analysis and Medical Imaging Group, Department of Applied Physics, University of Eastern Finland, Kuopio, Finland) to calculate the following heart rate variability parameters in the time domain [47]: SD of IBIs (SDNN [ms]) and root mean squared successive differences between IBIs (RMSSD [ms]). The mean HR, IBI, SDNN, and RMSSD were calculated during B_1_, B_3_, B_4_, B_6_, B_7_, and B_9_ (one-minute duration each) (cf. Figure 2).

### Statistical analysis

We checked normal distributions of all parameters by Shapiro–Wilk tests [48] and visually inspected histograms, skewness, and kurtosis. The fatigue parameters RMS_SLOPE_ and MPF_SLOPE_ were normally distributed, all other parameters showed a light to strong positive skew. Descriptive and muscular fatigue data are presented as means with standard deviations and muscular activity, upper body postures and heart rate and variability are presented as medians with interquartile ranges and as boxplots. Statistical analyses were performed using IBM SPSS Statistics for Windows (V28.0.0.0, IBM Corp., Armonk, NY, USA) and statistical significance was accepted when *p* < 0.05.

We performed generalized estimating equations (GEE) with exchangeable correlation matrices and linear scale responses to test the within-subject effects of condition (three levels: no, passive, active breaks) on RMS_SLOPE_ and MPF_SLOPE_. We performed generalized estimating equations (GEE) with exchangeable correlation matrices and inverse Gaussian scale responses to test the within-subject effects of condition (three levels: no, passive, active breaks) and time (six levels: B_1_, B_3_, B_4_, B_6_, B_7_, B_9_) on the following parameters: RMS_STATIC_, RMS_MEDIAN_, RMS_PEAK_, angle_MEDIAN_, HR, IBI, SDNN, RMSSD. In case of significant main or interaction effects, we performed Šidák for post hoc pairwise comparisons.

We calculated effect sizes for main or interaction effects using Cohen’s index *w* and for pairwise comparisons using Cohen’s *d* (average SD of both comparators as standardizer) [49] and interpreted them as small (*w* ≥ 0.1; *d* ≥ 0.2), medium (*w* ≥ 0.3; *d* ≥ 0.5), or large (*w* ≥ 0.5; *d* ≥ 0.8) [50].

## Results

Results for muscular fatigue are presented in Table 2, for muscular activity in Table 3, and for posture, heart rate, and heart rate variability in Table 4. Medians and interquartile ranges for muscular activity, posture, heart rate, and heart rate variability are provided as Supplemental Digital Content 3.

**Table 2:** Muscular fatigue: slopes of RMS (%MVE/min) and MPF (Hz/min) of the various muscles provided as mean (SD) for each experimental condition (without breaks, passive breaks, active breaks)

		Condition		
		Without	Passive	Active
ESR	RMS_SLOPE_	− 0.005 (0.041)	− 0.020 (0.081)	− 0.004 (0.014)
	MPF_SLOPE_	0.147 (0.446)	0.069 (0.335)	0.052 (0.205)
ESL	RMS_SLOPE_	− 0.002 (0.044)	− 0.009 (0.033)	0.004 (0.037)
	MPF_SLOPE_	0.024 (0.134)	− 0.075 (0.268)	0.017 (0.090)
TDR	RMS_SLOPE_	0.023 (0.034)	0.012 (0.034)	0.001 (0.038)
	MPF_SLOPE_	− 0.002 (0.034)	− 0.026 (0.053)	− 0.009 (0.053)
TDL	RMS_SLOPE_	0.020 (0.028)	0.023 (0.053)	0.008 (0.032)
	MPF_SLOPE_	0.012 (0.063)	0.004 (0.097)	0.002 (0.040)
DA	RMS_SLOPE_	0.009 (0.018)	0.002 (0.025)	0.000 (0.026)
	MPF_SLOPE_	− 0.011 (0.060)	− 0.002 (0.097)	− 0.026 (0.063)
ED	RMS_SLOPE_	0.014 (0.044)	0.010 (0.038)	0.003 (0.029)
	MPF_SLOPE_	0.003 (0.088)	− 0.016 (0.084)	− 0.030 (0.065)
FCR	RMS_SLOPE_	− 0.008 (0.026)	0.003 (0.015)	0.000 (0.014)
	MPF_SLOPE_	− 0.040 (0.195)	0.019 (0.192)	− 0.042 (0.089)

*ESR* erector spinae right, *ESL* erector spinae left, *TDR* trapezius descendens right, *TDL* trapezius descendens left, *DA* deltoid anterior, *ED* extensor digitorum, *FCR* flexor carpi radialis, *RMS* root mean square, *MPF* median power frequency, *MVE* maximal voluntery exertion

**Table 3:** Muscular activity: statistical results of the GEE and effect size index *w* for the main and interaction factors

		Condition			Time			Condition × Time		
		Wald χ^2^ (df)	*p*	*w*	Wald χ^2^ (df)	*p*	*w*	Wald χ^2^ (df)	*p*	*w*
ESR	RMS_STATIC_	2.534 (2)	0.282	0.084	23.012 (5)	0.000*	0.253	19.429 (10)	0.035	0.232
	RMS_MEDIAN_	3.186 (2)	0.203	0.094	17.092 (5)	0.004*	0.218	6.055 (10)	0.811	0.130
	RMS_PEAK_	2.874 (2)	0.238	0.089	11.164 (5)	0.048*	0.176	19.134 (10)	0.039*	0.231
ESL	RMS_STATIC_	0.232 (2)	0.890	0.025	23.645 (5)	0.000*	0.257	6.354 (10)	0.785	0.133
	RMS_MEDIAN_	0.271 (2)	0.873	0.027	20.093 (5)	0.001*	0.237	9.030 (10)	0.529	0.159
	RMS_PEAK_	0.365 (2)	0.833	0.032	14.955 (5)	0.011*	0.204	17.996 (10)	0.055	0.224
TDR	RMS_STATIC_	0.007 (2)	0.996	0.004	17.614 (5)	0.003*	0.216	20.309 (10)	0.026*	0.232
	RMS_MEDIAN_	0.020 (2)	0.990	0.007	20.301 (5)	0.001*	0.232	14.511 (10)	0.151	0.196
	RMS_PEAK_	0.003 (2)	0.999	0.003	25.610 (5)	0.000*	0.260	15.987 (10)	0.100	0.206
TDL	RMS_STATIC_	1.545 (2)	0.462	0.064	33.013 (5)	0.000*	0.298	10.820 (10)	0.372	0.171
	RMS_MEDIAN_	1.569 (2)	0.456	0.065	26.854 (5)	0.000*	0.269	16.393 (10)	0.089	0.210
	RMS_PEAK_	1.957 (2)	0.376	0.073	32.640 (5)	0.000*	0.296	32.389 (10)	0.000*	0.295
DA	RMS_STATIC_	0.610 (2)	0.737	0.040	4.090 (5)	0.537	0.104	25.931 (10)	0.004*	0.262
	RMS_MEDIAN_	2.171 (2)	0.338	0.076	4.832 (5)	0.437	0.113	22.180 (10)	0.014*	0.242
	RMS_PEAK_	3.958 (2)	0.138	0.102	3.119 (5)	0.682	0.091	41.551 (10)	0.000*	**0.332*†***
ED	RMS_STATIC_	0.583 (2)	0.747	0.039	30.751 (5)	0.000*	0.285	22.933 (10)	0.011*	0.246
	RMS_MEDIAN_	0.702 (2)	0.704	0.043	23.319 (5)	0.000*	0.248	26.206 (10)	0.003*	0.263
	RMS_PEAK_	0.982 (2)	0.612	0.051	9.511 (5)	0.090	0.159	30.603 (10)	0.001*	0.285
FCR	RMS_STATIC_	0.053 (2)	0.974	0.012	10.233 (5)	0.069	0.165	18.579 (10)	0.046*	0.222
	RMS_MEDIAN_	0.130 (2)	0.937	0.019	5.921 (5)	0.314	0.125	17.304 (10)	0.068	0.214
	RMS_PEAK_	0.778 (2)	0.678	0.045	14.338 (5)	0.014*	0.195	21.492 (10)	0.018*	0.238

*ESR* erector spinae right, *ESL* erector spinae left, *TDR *trapezius descendens right, *TDL* trapezius descendens left, *DA* deltoid anterior, *ED* extensor digitorum, *FCR* flexor carpi radialis, *RMS* root mean square, *statistically significant (*p* < 0.05) are given in bold, †medium effect size (χ2 ≥ 0.3) is given in bold

**Table 4:** Posture, heart rate, heart rate variability: statistical results of the GEE and effect size index *w* for the main and interaction factors

	Condition			Time			Condition × Time		
	Wald χ^2^ (df)	*p*	*w*	Wald χ^2^ (df)	*p*	*w*	Wald χ^2^ (df)	*p*	*w*
NF	1.144 (2)	0.564	0.055	21.897 (5)	**0.001**	0.241	30.291 (10)	0.001	0.283
NLF	0.085 (2)	0.958	0.015	8.337 (5)	0.139	0.149	7.647 (10)	0.663	0.142
TK	1.067 (2)	0.586	0.053	16.249 (5)	0.006*	0.207	53.562 (10)	0.000*	**0.376*†***
LL	1.138 (2)	0.566	0.055	10.511 (5)	0.062	0.167	8.410 (10)	0.589	0.149
HR	5.190 (2)	0.075	0.117	9.957 (5)	0.076	0.162	15.479 (10)	0.116	0.202
IBI	5.485 (2)	0.064	0.120	7.824 (5)	0.166	0.144	16.552 (10)	0.085	0.209
SDNN	8.517 (2)	0.014*	0.150	29.729 (5)	0.000*	0.280	35.852 (10)	0.000*	**0.308*†***
RMSSD	2.830 (2)	0.243	0.087	9.654 (5)	0.086	0.160	35.102 (10)	0.000*	**0.305*†***

*NF* neck flexion, *NLF* neck lateral flexion, *TK* thoracic kyphosis, *LL* lumbar lordosis, *HR* heart rate, *IBI* interbeat interval, *SDNN* SD of IBIs, *RMSSD* root mean squared successive differences between IBIs, *statistically significant (*p* < 0.05) are given in bold; †medium effect size (χ2≥0.3) are given in bold

### Muscular fatigue

**Fig. 3:**
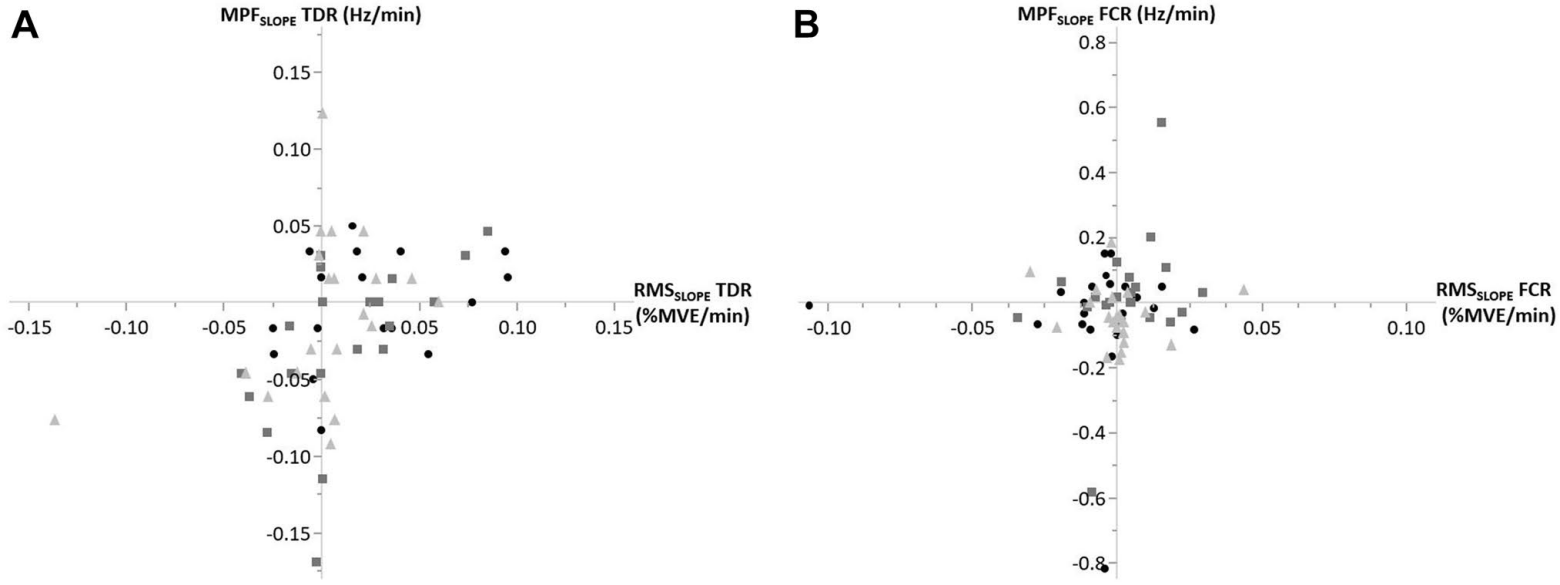
JASA plots displaying tendencies for muscular fatigue (lower right quadrant) in some subjects across some conditions (black dots, without; dark gray squares, passive; light gray triangles, active) for the trapezius descendens right (TDR, left plot) and flexor carpi radialis (FCR, right plot)

There were a few individual tendencies pointing to localized muscular fatigue (Fig. 3). In particular, the RMS of the TDR showed to statistically significant more signs of muscular fatigue (*p* < 0.05; *d* =  − 0.598) in the condition without breaks (slope 0.023) compared to active breaks (0.001). However, this significantly increased RMS was not accompanied by a significantly decreased MPF of the TDR. Overall, among most subjects and conditions, slopes of the RMS and MPF were spread throughout the JASA plots. This means that neither the control nor the intervention conditions led to clear signs of localized muscular fatigue, with an exception of the right shoulder muscle that tended to show fewer signs of muscular fatigue in the active break condition compared to the control.

### Muscular activity

None of the muscular activity levels showed a statistically significant effect of *Condition*.

Statistically significant main effects of *Time* were found for RMS_STATIC_ (*p* < 0.001; *w* = 0.253), RMS_MEDIAN_ (*p* < 0.01; *w* = 0.218), and RMS_PEAK_ (*p* < 0.05; *w* = 0.176) of ESR. Both RMS_STATIC_ (*MD* =  − 0.449%MVE; *p* = 0.045; *d* =  − 0.199) and RMS_MEDIAN_ (*MD* =  − 0.493%MVE; *p* = 0.033; *d* =  − 0.137) decreased from B_3_ to B_7_. RMS_PEAK_ of the ESR showed a statistically significant interaction effect of *Condition* × *Time* (*p* < 0.05; *w* = 0.231) without significant post hoc pairwise comparisons.

Statistically significant main effects of *Time* were found for RMS_STATIC_ (*p* < 0.001; *w* = 0.257), RMS_MEDIAN_ (*p* < 0.01; *w* = 0.237), and RMS_PEAK_ (*p* < 0.05; *w* = 0.204) of ESL. For all three levels, muscular activity decreased on average from B_1_ to B_7_ with − 0.777 (*p* < 0.001; *d* =  − 0.267), − 0.753 (*p* = 0.001; *d* =  − 0.225), and − 0.694%MVE (*p* = 0.009; *d* =  − 0.178), respectively.

**Fig. 4:**
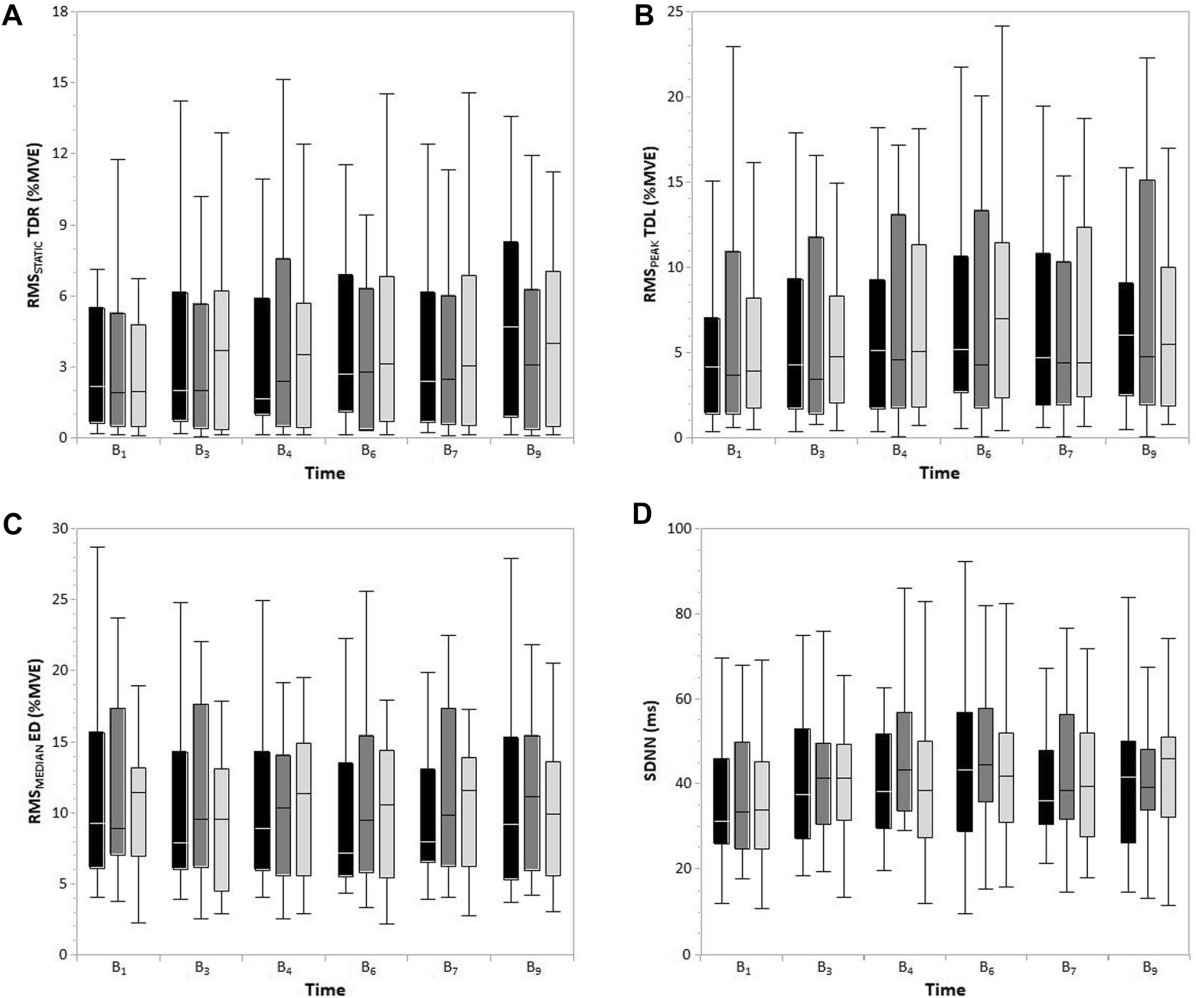
Boxplots displaying minimum, 1st quantile, median, 3rd quantile and maximum of the RMS_STATIC_ of the right trapezius descendens (TDR, left upper corner), RMS_PEAK_ of the left trapezius descendens (TDL, right upper corner), RMS_MEDIAN_ of the extensor digitorum (ED, left lower corner), and SDNN (right lower corner). The three conditions are displayed with black (without breaks), dark gray (passive breaks), or light gray filling (active breaks)

Statistically significant main effects of *Time* were found for RMS_STATIC_ (*p* < 0.01; *w* = 0.216), RMS_MEDIAN_ (*p* < 0.01; *w* = 0.232) and RMS_PEAK_ (*p* < 0.001; *w* = 0.260) of TDR. RMS_STATIC_ (Fig. 4) increased from B_1_ to B_6_ (*MD* = 1.178%MVE; *p* = 0.037; *d* = 0.356) and B_9_ (*MD* = 1.288%MVE; *p* = 0.009; *d* = 0.391); RMS_MEDIAN_ increased from B_1_ (*MD* = 1.526%MVE; *p* = 0.008; *d* = 0.349) and B_7_ (*MD* = 0.667%MVE; *p* = 0.006; *d* = 0.138) to B_9_; RMS_PEAK_ increased from B_1_ to B_4_ (*MD* = 1.445%MVE; *p* = 0.043; *d* = 0.249). RMS_STATIC_ showed a statistically significant interaction effect of *Condition* × *Time* (*p* < 0.05; *w* = 0.232), where activity at B_9_ was higher than at B_1_ (*MD* = 2.025%MVE; *p* = 0.016; *d* = 0.617), B_4_ (*MD* = 1.516%MVE; *p* = 0.003; *d* = 0.414), and B_7_ (*MD* = 1.095%MVE; *p* = 0.004; *d* = 0.281) in the condition without breaks.

Statistically significant main effects of Time were found for RMSSTATIC (*p* < 0.001; w = 0.298), RMSMEDIAN (*p* < 0.001; *w* = 0.269), and RMSPEAK (*p* < 0.001; w = 0.296) of TDL. RMSSTATIC increased from B1 to B4 (*MD* = 0.875%MVE; *p* = 0.011; *d* = 0.266), B6 (*MD* = 0.824%MVE; *p* = 0.014; *d* = 0.271) and B9 (*MD* = 1.240%MVE; *p* < 0.001; *d* = 0.355) and from B3 to B9 (*MD* = 0.901%MVE; *p* = 0.002; d = 0.256); RMSMEDIAN increased from B1 (*MD* = 1.435%MVE; *p* = 0.001; *d* = 0.300) and B3 (*MD* = 1.064%MVE; *p* < 0.001; *d* = 0.220) to B9; RMSPEAK (Fig. 4) increased from B1 (*MD* = 1.662%MVE; *p* = 0.017; *d* = 0.264) and B3 (*MD* = 1.227%MVE; *p* < 0.001; *d* = 0.192) to B6 and from B3 to B9 (*MD* = 1.357%MVE; *p* < 0.001; *d* = 0.208). RMSPEAK showed a statistically significant interaction effect of Condition × Time (*p* < 0.001; *w* = 0.295), without significant post hoc pairwise comparisons.

DA showed statistically significant interaction effects of *Condition* × *Time* for RMS_STATIC_ (*p* < 0.01; *w* = 0.262), RMS_MEDIAN_ (*p* < 0.05; *w* = 0.242), and RMS_PEAK_ (*p* < 0.001; *w* = 0.332), without significant post hoc pairwise comparisons.

Statistically significant main effects of *Time* were found for RMS_STATIC_ (*p* < 0.001; *w* = 0.285) and RMS_MEDIAN_ (*p* < 0.001; *w* = 0.248) of ED. RMS_STATIC_ decreased from B_1_ to B_3_ (*MD* =  − 1.662%MVE; *p* < 0.001; *d* =  − 0.448), B_4_ (*MD* =  − 0.858%MVE; *p* = 0.015; *d* =  − 0.218), B_6_ (*MD* =  − 1.500%MVE; *p* < 0.001; *d* =  − 0.384) and B_7_ (*MD* =  − 0.964%MVE; *p* = 0.002; *d* =  − 0.247) and increased from B_3_ o B_4_ (*MD* = 0.804%MVE; *p* = 0.016; *d* = 0.233); RMS_MEDIAN_ (Fig. 4) decreased from B_1_ to B_3_ (*MD* =  − 0.836%MVE; *p* = 0.002; *d* =  − 0.211). Statistically significant interaction effects of *Condition* × *Time* were found for RMS_STATIC_ (*p* < 0.05; *w* = 0.246), RMS_MEDIAN_ (*p* < 0.01; *w* = 0.263), and RMS_PEAK_ (*p* < 0.01; *w* = 0.285). RMS_MEDIAN_ increased from B_3_ to B_4_ (*MD* = 1.370%MVE; *p* = 0.022; *d* = 0.221) for the condition with active breaks; RMS_PEAK_ decreased from B_1_ to B_4_ (*MD* =  − 3.394%MVE; *p* = 0.031; *d* =  − 0.429) for the condition with passive breaks.

For FCR, a statistically significant main effect of *Time* was found for RMS_PEAK_ (*p* < 0.05; *w* = 0.195) and statistically significant interaction effects of *Condition* × *Time* were found for RMS_STATIC_ (*p* < 0.05; *w* = 0.222) and RMS_PEAK_ (*p* < 0.05; *w* = 0.238). None of the post hoc pairwise comparisons were statistically significant.

### Upper body postures

#### None of the joint angles showed a statistically significant effect of *Condition*

NF showed a statistically significant main effect of *Time* (*p* < 0.01; *w* = 0.241); NF increased from B_3_ to B_7_ (*MD* = 0.359°; *p* = 0.045; *d* = 1.710). TK showed a statistically significant main effect of *Time* (*p* < 0.01; *w* = 0.207) and interaction effect of *Condition* × *Time* (*p* < 0.001; *w* = 0.376), but post hoc pairwise comparisons were not statistically significant.

### Heart rate and variability

HR showed no statistically significant main or interaction effects. The HRV parameter SDNN (Fig. 4) showed a statistically significant main effect of *Condition* (*p* < 0.05; *w* = 0.150), but without significant post hoc pairwise comparisons. SDNN also showed a statistically significant main effect of *Time* (*p* < 0.001; *w* = 0.280), which increased from B_1_ to B_4_ (*MD* = 7.536 ms; *p* = 0.007; *d* = 0.471), B_6_ (*MD* = 10.126 ms; *p* < 0.001; *d* = 0.565), B_7_ (*MD* = 7.452 ms; *p* = 0.035; *d* = 0.458), and B_9_ (*MD* = 7.399 ms; *p* = 0.027; *d* = 0.453). SDNN also showed a statistically significant interaction effect of *Condition* × *Time* (*p* < 001; *w* = 0.308); B_4_ in the condition with passive breaks was higher than B_1_ in the conditions without breaks (*MD* = 13.767 ms; *p* = 0.004; *d* = 0.818) and with active breaks (*MD* = 13.561 ms; *p* = 0.015; *d* = 0.777). The HRV parameter RMSSD showed a statistically significant interaction effect of *Condition* × *Time* (*p* < 001; *w* = 0.305), but without statistically significant post hoc pairwise comparisons.

## Discussion

Performing surgeries laparoscopically instead of open is beneficial for the patient’s well-being [6–8]; however, surgeons are put at higher risk for developing musculoskeletal complaints and disorders due to the constrained and static working postures associated with laparoscopy [2–5]. This study evaluated the organizational measure of passive and active intraoperative work breaks compared to no breaks during simulated laparoscopy with respect to muscular fatigue, muscular activity, upper body posture, heart rate, and heart rate variability. Muscular fatigue tended to be less in the intervention conditions compared to the control condition. Also, the heart rate variability metric SDNN tended to be lower in the control condition without breaks compared to the intervention conditions with breaks. Both tendencies reflects to an effect in favor of intraoperative (passive/active) work breaks, because less muscular fatigue [51] and more heart rate variability [47] may reduce the risk associated with work-related the physical stress response. No (tendencies of) statistically significant effects of work break intervention were detected for muscular activity, joint angles or heart rate. The factor time had a statistically significant effect mainly on the muscular activity parameters, which generally tended to increase over time irrespective of the experimental condition (Fig. 4).

### Muscular fatigue

A “less-is-better”-principle applies to muscular fatigue, which is an acknowledged precursor of developing musculoskeletal disorders [51]. In previous studies, especially the dominant trapezius showed to be vulnerable to localized muscular fatigue, since the trapezius is often the main affected muscle in the lead up to musculoskeletal complaints [17, 52]. Although the right (dominant) trapezius muscle tended to fatigue more in the control condition compared to the intervention conditions, this was based on its RMS only. Therefore, only the dominant shoulder muscles might benefit from intraoperative work breaks, whereas for all other muscles we could not detect comparable tendencies in the 1.5-h laparoscopy as simulated in the current study (Fig. 3; Table 2).

### Muscular activity

Related to muscular fatigue is the risk that prolonged activity of small, single muscle fibers may cause degenerative muscular changes, even with very low levels of static muscular activity [53]. However, over time, only the bilateral trapezius muscle increased its static, median and peak level over time. Note that the left (i.e., non-dominant) and right (i.e., dominant) body sides for the forearm extensors showed muscular activity fluctuation over time of < 1%MVC. In line with the tendency for muscular fatigue, muscular activity on the dominant right side also appears to increase slightly more over time compared to the non-dominant left side, suggesting that the dominant shoulder may be more susceptible to developing future musculoskeletal disorders [54]. However, the trapezius muscles as well as the other back and forearm muscles remained below the 30%MVC-threshold-limit-value for laparoscopic work (estimated hand activity level of 6) [55]. This is in line with a recent study that documented traditional laparoscopic work, where the peak muscular activity level did not exceed 15%MVC [56]. Since the current study could not identify significant effects of both intervention conditions on any of the muscular activity levels, a potential protective effect of passive or active work breaks with respect to the risk of developing musculoskeletal disorders could not be identified [57]. Although upper threshold limit values for hand activities are set, future research remains challenged for providing lower threshold limit values for hand activities, including the duration of static work periods with respect to recovery in light of the Cinderella hypothesis and muscular fatigue [58, 59]. Some argue that a lower threshold limit value of 2 to 5%MVC should not be exceeded [60]; if this limit value is to be validated, laparoscopic work has to be considered risky considering that static muscular activity levels exceeded 2%MVC in several muscles [56].

### Upper body postures

Neck flexion ≥ 20° [61], and more thoracic kyphosis [62] and limited lumbar lordosis [63] are identified as potential risk factors for developing neck-related musculoskeletal disorders or low back pain, respectively. The current study found a statistically significant increase of the neck flexion angle over time; however, median neck flexion angles were in the range of 4.4 to 7.2° and would not be considered risky in terms of developing musculoskeletal disorders. None of the joint angles investigated were influenced by the implementation of work breaks to a significant extent.

### Heart rate and variability

An increased heart rate and reduced heart rate variability have been associated with work-related physical and mental stress, particularly reflecting the dominant role of the sympathetic nervous system during work [47, 64]. The HRV parameter SDNN, a parameter recommended for physical stress, tended to be lower in the condition without work breaks (38 ms) than in the conditions with passive (40 ms) and active work breaks (39 ms). However, this tendency is too minimal to detect a statistically significant or relevant influence of the implementation of work breaks on the sympathetic activity response to laparoscopic surgery.

### Study implications and limitations

The reason for investigating work break interventions comes from promising results identified among office workers, who predominantly perform computer work. Recent studies showed that especially back and neck pain are reduced when providing active work breaks [65, 66]. Also for intensive care unit nurses, active work breaks are being recommended [67]. Since several field pilot studies have been performed among laparoscopic surgeons with the main outcome of perceived musculoskeletal discomfort, the current exploratory study aimed to provide insights into the acute effects of implementing passive and active work breaks in a simulated environment on physical strain and stress parameters of gynecological surgeons. We could not identify significant effects of both types of work breaks, only tendencies in favor of a reduced risk for work-related musculoskeletal disorders and physical stress response. Considering previous and the current study results, a future field feasibility study should provide insights into the acceptability and practicability of intraoperative active work breaks. When promising, a follow-up cluster randomized controlled trial should reveal the true effectiveness with respect to different outcome measures of intraoperative active work breaks.

Although the required sample size was reached (note that the calculation was based on rating of perceived discomfort data), the current study has some limitations that should be mentioned. First, we focused on the principal surgeon only, without considering potential effects work breaks may have on the remaining surgical team including assistant surgeons, anesthesiologists, and nurses [25]. Second, we focused on physical strain and stress parameters only, whereas outcomes such as discomfort, performance, and mental status may be interesting to investigate as well. Another manuscript will report on some of these outcomes. Third, due to the laboratory setting and the simulated nature of the study set-up, we are not able to provide direct associations with work-related musculoskeletal disorders. Fourth, the analysis of HRV was based on only one minute in each case, i.e., shorter than recommended in the current German guideline [47]. Finally, the way we have analyzed and reported outcomes may be improved in future studies by providing insights in the accumulated time surgeons spent working in particular muscular loads and static postures (i.e., joint angles). Configuring such an extensive profile is possible by applying the Exposure Variation Analysis [68].

## Conclusion

This randomized, controlled cross-over laboratory trial demonstrated tendencies in favor of 2.5-min intraoperative work breaks presented after 30-min work blocks, including the tendencies of decreased shoulder muscular fatigue and increased heart rate variability (SDNN). The tentative results may point toward a potential relieving effect on the risk for developing musculoskeletal disorders and physical stress responses. Note that the tendencies for potential positive effects of intraoperative work breaks as detected in the here investigated simulated laparoscopy are, in a real laparoscopic surgery, highly dependent on the complexity and duration of the surgery, composition of the surgical team (enough skilled surgeons present), sequence of the surgery (first surgery or after several surgeries), and physical condition of the surgeon. The effect of passive and active breaks on perceived musculoskeletal discomfort and performance is to be evaluated in a follow-up manuscript. Following the outcomes of this study and taking in consideration the context of a real surgical laparoscopy, a field feasibility study should validate the acceptability and practicability of intraoperative active work breaks during laparoscopic procedures, after which a cluster randomized controlled trial can assess its effectiveness.

## Supplementary Information

Below is the link to the electronic supplementary material.

Supplementary file1 (DOCX 13 KB)

Supplementary file2 (DOCX 14 KB)

Supplementary file3 (DOCX 17 KB)

## Declarations

### Amendment to study protocol

We did not include order of the experimental condition as a confounding factor, because it was randomized across subjects.
