# Supplementary material for: Intraoperative active and passive breaks during minimally invasive surgery influence upper extremity physical strain and physical stress response—A controlled, randomized cross-over, laboratory trial
Source: Surg Endosc. 2023 Apr 21;37(8):5975–88. doi: 10.1007/s00464-023-10042-9 (PMC10120511; doi:10.1007/s00464-023-10042-9)
Supplement: Supplementary file 1 — Supplementary file1 (DOCX 13 KB) [file 464_2023_10042_MOESM1_ESM.docx]

**SUPPLEMENTAL DIGITAL CONTENT 1**

**Speech protocol for the audio recording of the mobility and stretching exercises for the active work breaks** *(translated from German)*

- Starting position that you take during the entire active break:
  Take a hip-wide standing position and clasp your hands in front of your chest.
- Exercise 1:
  Start walking on the spot without lifting your toes off the floor while slightly rotating your upper body.
- Exercise 2:
  Move your hips alternately from left to right.
- Exercise 3:
  Alternately move your pelvis back and forth. This alternately leads to a light hollow cross or rounded back.
- Exercise 4:
  Bring your hips forward and push your sternum up and pull your shoulders slightly back. Hold the end position for a few seconds.
- Exercise 5:
  Make small backward circular movements in your shoulders.
- Exercise 6:
  Make your neck long by making a light double chin. Hold the end position for a few seconds.
- Exercise 7:
  Turn your head (without force) alternately from left to right.
- Exercise 8:
  Start walking on the spot without lifting your toes off the floor while slightly rotating your upper body.
