# Supplementary material for: Intraoperative active and passive breaks during minimally invasive surgery influence upper extremity physical strain and physical stress response—A controlled, randomized cross-over, laboratory trial
Source: Surg Endosc. 2023 Apr 21;37(8):5975–88. doi: 10.1007/s00464-023-10042-9 (PMC10120511; doi:10.1007/s00464-023-10042-9)
Supplement: Supplementary file 2 — Supplementary file2 (DOCX 14 KB) [file 464_2023_10042_MOESM2_ESM.docx]

**SUPPLEMENTAL DIGITAL CONTENT 2**

**Maximal voluntary contractions for each muscle**

- Erector spinae longissimus lumbalis (at vertebra L3): The subject lay prone with the upper body and hips (hip bones) off the bench and the legs fixed with straps, performing maximal hip extension against a barrier while keeping the body horizontal and the arms crossed in front of the chest (modified Biering-Sørensen test).
- Trapezius descendens: The subject stood upright, feet hip-width apart, arms in 90◦ abduction but slightly in the frontal plane, elbows extended but not overstretched, while performing maximal arm abduction while a ring with chain mounted on the floor was placed over the upper arm just below the electrodes of the deltoid acromialis.
- Deltoid acromialis: Similar procedure as for trapezius descendens.
- Extensor digitorum: The subject stood upright, lower arm from wrist to elbow resting on a height-adjustable table with the hand palm facing down, hand clenched into a fist, while performing maximal extension against resistance of one of the experimenters.
- Flexor carpi radialis: The subject stood upright, lower arm from wrist to elbow resting on a height-adjustable table with the hand palm facing up, hand clenched into a fist, while performing maximal extension against resistance of one of the experimenters.
