# Supplementary material for: Intraoperative active and passive breaks during minimally invasive surgery influence upper extremity physical strain and physical stress response—A controlled, randomized cross-over, laboratory trial
Source: Surg Endosc. 2023 Apr 21;37(8):5975–88. doi: 10.1007/s00464-023-10042-9 (PMC10120511; doi:10.1007/s00464-023-10042-9)
Supplement: Supplementary file 3 — Supplementary file3 (DOCX 17 KB) [file 464_2023_10042_MOESM3_ESM.docx]

**SUPPLEMENTAL DIGITAL CONTENT 3**

**Median values with interquartile ranges for parameters of muscular activity, posture, heart rate and heart rate variability**

**Table SDC3.1**. Muscular activity: median value (IQR) for the static, median and peak levels of muscular activity for the various muscles per experimental condition (without breaks, passive breaks, active breaks).

|  | | **Condition** | | |
| --- | --- | --- | --- | --- |
|  |  | **Without** | **Passive** | **Active** |
| **ESR** | **RMS_STATIC_** | 1.807 (2.373) | 2.267 (2.399) | 1.974 (1.120) |
|  | **RMS_MEDIAN_** | 3.012 (3.313) | 3.255 (2.469) | 2.763 (1.945) |
|  | **RMS_PEAK_** | 4.217 (3.772) | 4.380 (2.823) | 3.947 (2.013) |
| **ESL** | **RMS_STATIC_** | 5.176 (4.363) | 5.331 (4.049) | 5.080 (4.371) |
|  | **RMS_MEDIAN_** | 6.314 (4.825) | 6.304 (4.144) | 6.311 (4.693) |
|  | **RMS_PEAK_** | 7.586 (5.420) | 7.546 (4.316) | 7.523 (5.203) |
| **TDR** | **RMS_STATIC_** | 2.611 (5.592) | 2.525 (5.541) | 3.215 (5.234) |
|  | **RMS_MEDIAN_** | 3.926 (7.326) | 4.283 (6.923) | 5.171 (7.655) |
|  | **RMS_PEAK_** | 6.265 (8.583) | 6.798 (7.881) | 7.733 (9.636) |
| **TDL** | **RMS_STATIC_** | 1.901 (3.734) | 1.268 (5.302) | 2.147 (4.486) |
|  | **RMS_MEDIAN_** | 3.730 (5.549) | 2.520 (7.030) | 3.245 (6.583) |
|  | **RMS_PEAK_** | 5.178 (7.394) | 4.122 (10.177) | 4.575 (8.309) |
| **DA** | **RMS_STATIC_** | 1.416 (1.395) | 1.472 (1.461) | 1.451 (1.303) |
|  | **RMS_MEDIAN_** | 2.394 (2.484) | 2.488 (2.311) | 2.794 (2.342) |
|  | **RMS_PEAK_** | 4.036 (3.815) | 3.745 (2.958) | 4.829 (3.619) |
| **ED** | **RMS_STATIC_** | 4.137 (4.674) | 5.082 (6.376) | 5.572 (4.313) |
|  | **RMS_MEDIAN_** | 8.012 (8.416) | 9.869 (10.183) | 10.639 (7.635) |
|  | **RMS_PEAK_** | 14.564 (12.988) | 17.004 (13.176) | 17.146 (9.455) |
| **FCR** | **RMS_STATIC_** | 1.167 (0.954) | 1.271 (0.929) | 1.084 (0.886) |
|  | **RMS_MEDIAN_** | 2.707 (2.140) | 2.575 (2.658) | 2.558 (2.642) |
|  | **RMS_PEAK_** | 6.599 (5.644) | 5.265 (4.742) | 6.275 (5.702) |
| *ESR, erector spinae right; ESL, erector spinae left; TDR, trapezius descendens right; TDL, trapezius descendens left; DA, deltoid anterior; ED, extensor digitorum; FCR, flexor carpi radialis, RMS, root mean square; MPF, median power frequency.* | | | | |

**Table SDC3.2**. Posture, heart rate and heart rate variability: median (IQR) for the various parameters per experimental condition (without breaks, passive breaks, active breaks.

|  | **Condition** | | |
| --- | --- | --- | --- |
|  | **Without** | **Passive** | **Active** |
| **NF** | −7.225 (16.663) | −4.425 (10.813) | −5.875 (12.900) |
| **NLF** | 5.400 (8.525) | 5.800 (10.675) | 4.425 (8.563) |
| **TK** | 3.725 (8.725) | 3.000 (6.538) | 2.275 (7.550) |
| **LL** | 1.675 (8.975) | −0.575 (7.050) | 2.100 (7.463) |
| **HR** | 82.807 (17.386) | 81.583 (18.169) | 82.728 (17.963) |
| **IBI** | 724.572 (155.319) | 735.449 (180.669) | 725.271 (156.565) |
| **SDNN** | 37.980 (20.528) | 40.045 (19.613) | 39.000 (21.548) |
| **RMSSD** | 20.415 (16.253) | 22.578 (15.318) | 20.114 (15.222) |
| *NF, neck flexion; NLF, neck lateral flexion; TK, thoracic kyphosis; LL, lumbar lordosis; HR, heart rate; IBI, interbeat interval; SDNN, SD of IBIs; RMSSD, root mean squared successive differences between IBIs.* | | | |
